# Anterior Hepatic Herniation: An Unusual Presentation of Abdominal Incisional Hernia

**DOI:** 10.7759/cureus.4066

**Published:** 2019-02-13

**Authors:** Eric O Then, Febin John, Andrew Ofosu, Vinaya Gaduputi

**Affiliations:** 1 Internal Medicine, Saint Barnabas Hospital, Bronx, USA; 2 Gastroenterology, Brooklyn Hospital Center, Affiliate of the Mount Sinai Hospital, Brooklyn, USA; 3 Gastroentrology, Brooklyn Hospital Center, Affiliate of the Mount Sinai Hospital, Brooklyn, USA; 4 Gastroenterology and Hepatology, Saint Barnabas Hospital, Bronx, USA

**Keywords:** ventral hernia, hepatic herniation, liver herniation, budd chiari syndrome, steatosis

## Abstract

Hepatic herniation through an abdominal incisional hernia is a rare phenomenon that has been seldom reported in the medical literature. When present, this may cause patients significant distress and is associated with complications such as hepatic encephalopathy and Budd-Chiari syndrome. Most cases can be managed conservatively through observation, but many cases require surgical intervention to preserve hepatic function. Our case consists of a 54-year-old man who presented with asymptomatic herniation of the left hepatic lobe through an abdominal incisional hernia.

## Introduction

Ventral hernia is a bulge through an opening in the abdominal wall, allowing internal organs such as bowel to be dislocated from its original position. When herniation occurs through a prior scar, it is called an incisional hernia. These events are not uncommon, most of them presenting with bowel in the hernial sac [1]. However, herniation of the liver through the abdominal wall is a rare occurrence. To our knowledge this is the sixth such case reported in the medical literature. Hepatic herniation through a defect in the diaphragm is a separate entity that mostly commonly presents in the pediatric population and is outside the scope of this discussion [2].

## Case presentation

**Figure 1:**
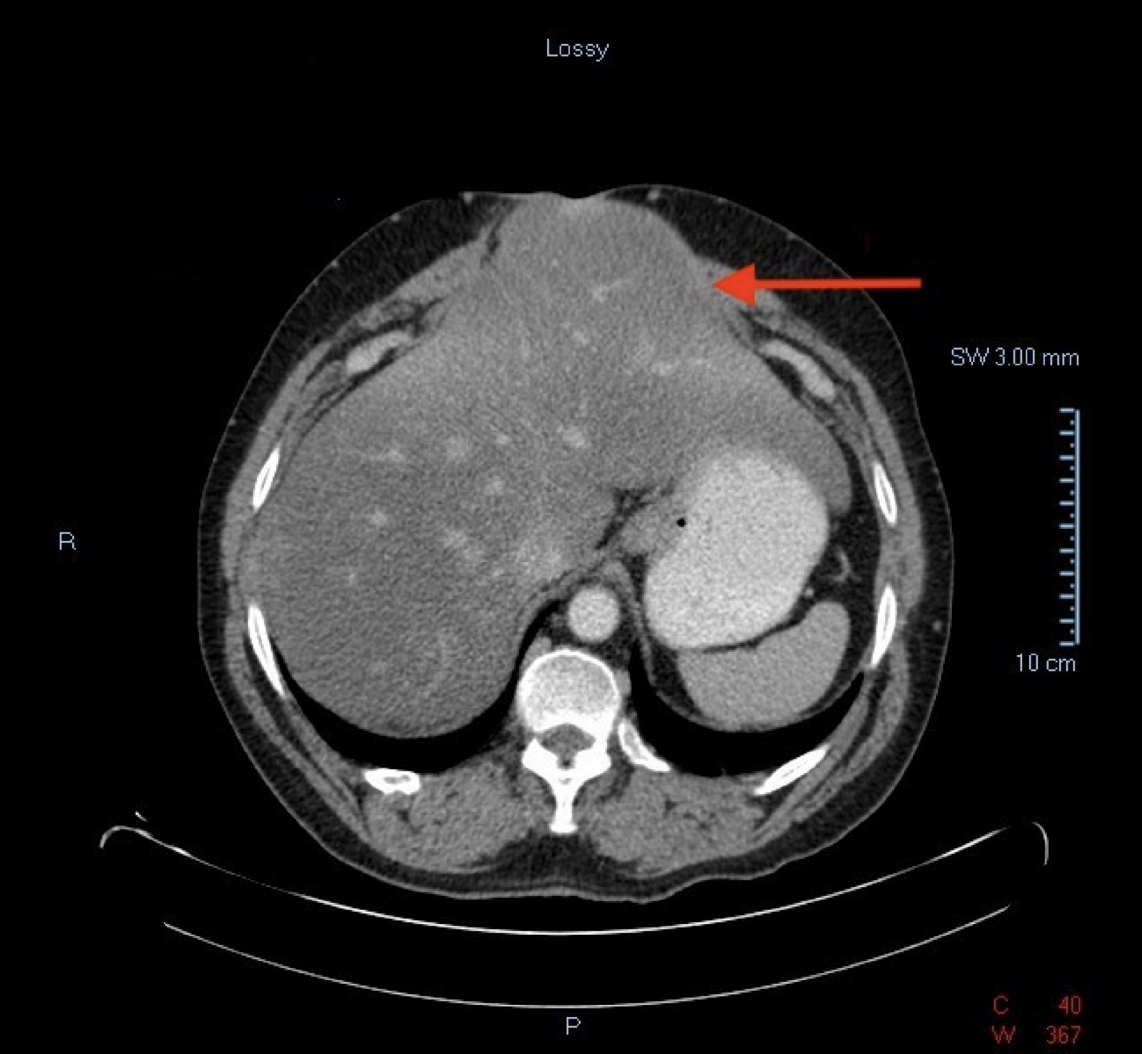
CT abdomen (axial view) showing herniation of the left hepatic lobe through an incisional abdominal hernia.

**Figure 2:**
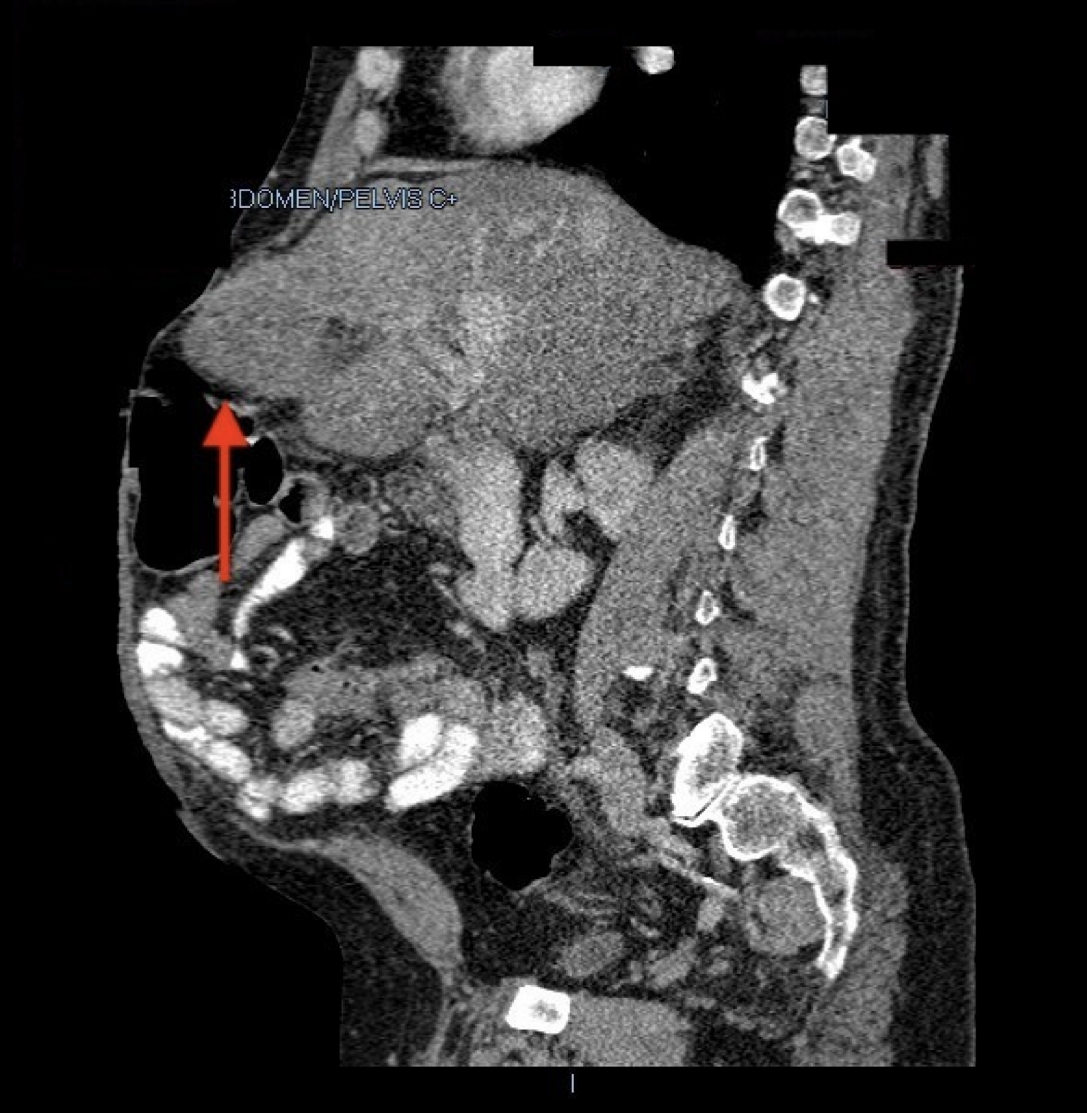
CT abdomen (sagittal view) showing herniation of the left hepatic lobe through an incisional abdominal hernia.

A 54-year-old Hispanic male with a medical history significant for chronic obstructive pulmonary disease (COPD), diabetes mellitus, alcohol abuse, heroin abuse (on methadone), hepatitis C, latent tuberculosis, and ventral hernia repair presented to our institution’s emergency department complaining of progressive shortness of breath for one month duration. The patient also complained of increased abdominal girth within the same period of time, which he stated worsened his shortness of breath. Initial workup included a chest X-ray showing left lower lobe atelectasis. Notable laboratory findings included hemoglobin: 12.6 g/dL, white blood cell count: 11.5 103/uL, platelet count: 208 103/uL, creatinine: 1.4 mg/dL, blood urea nitrogen: 24 mg/dL, alanine aminotransferase (ALT): 37 IU/L, aspartate aminotransferase (AST): 52 IU/L, total bilirubin: 0.5 mg/dL, and alkaline phosphatase: 72 IU/L. The patient was then admitted to the medical floor under the impression of COPD exacerbation. On admission, surgery was consulted to evaluate the patient’s increasing abdominal girth given his medical history of ventral hernia. An abdominal CT scan was done, which showed the anterior portion of the left hepatic lobe partially herniating through the ventral hernia accompanied by hepatic steatosis (Figures 1-2). Physical examination revealed the patient in respiratory distress, with a distended abdomen, a midline scar, and a lump protruding through the midline. Vital signs included a blood pressure of 145/95 mmHg, heart rate of 133 beats per minute, and respiratory rate of 30 breaths per minute. Due to the patient’s ongoing medical conditions, repair of the hernia was not done as an inpatient. During his hospital stay, the patient’s respiratory status improved and he was safely discharged home with a follow up appointment to the surgery clinic for hernia repair as an outpatient.

## Discussion

Herniation of the liver or its parts through the abdominal wall is a rare phenomenon that was first described by Adeonigbagbe et al. in the year 2000 [3]. Since then, there have been few cases reported in the medical literature. Isolated incisional hernias are not uncommon with incidence rates ranging from 2% to 20% per year [4]. Epidemiologic data are not available on hepatic herniation through the abdominal wall due to its rarity. Reported cases have occurred in association with abdominal incisional hernias, nonalcoholic steatohepatitis, coronary artery bypass grafting (CABG), or direct trauma to the abdomen [5-6].

Risk factors associated with the development of hepatic herniation through the abdominal wall include obesity, old age, poor nutrition, increased intra-abdominal pressure, smoking, weak abdominal wall, and postsurgical site infection [7]. In addition, intrinsic weaknesses of the abdominal wall and increases in intra-abdominal pressure predispose patients to develop ventral hernias and subsequently, herniation of the liver. Another theory postulated by Echo et al, suggests that congenital absence of the left or right triangular ligaments of the liver are possible risk factors. These structures are responsible for fixing the liver to the retroperitoneum and their absence in theory can result in anterior herniation of segments of the liver when coupled with the aforementioned risk factors [8]. In our patient, the triad of hepatic steatosis, history of previous abdominal surgery, and an increase in intra-abdominal pressure secondary to frequent COPD exacerbations were thought to be the cause of his hepatic herniation. 

Presenting symptoms of abdominal hepatic herniation include abdominal pain, nausea, vomiting, jaundice, dyspnea, confusion, and epigastric swelling [9]. It is often found incidentally on imaging of the abdomen. Clinical consequences of hepatic herniation can be severe and may vary based on which lobe of the liver is herniated. Herniation of the left hepatic lobe has been associated with incarceration of the liver within the hernial sac that can lead to hepatic encephalopathy and liver failure. In one reported case, the afflicted patient presented with increased hepatic transaminases, which manifested as flapping tremors and hepatic encephalopathy [10]. Herniation of the right hepatic lobe in contrast has been linked to Budd-Chiari syndrome . In that case, a 75-year-old woman presented with right hepatic lobe herniation after undergoing right partial nephrectomy 52 years prior. Although she was asymptomatic, CT scan confirmed the presence of a secondary Budd-Chiari syndrome [11]. It is important for a clinician to consider this complication given its associated morbidity and mortality.

Hepatic hernias should be suspected when a patient presents with epigastric bulging, but adequate diagnosis requires imaging studies such as CT scan. In our patient, CT scan was sufficient in demonstrating hepatic herniation through a previous abdominal incisional hernia. Once diagnosed, treatment of hepatic hernias can be a challenge. Currently there are no guidelines that dictate treatment and most cases can be managed conservatively. If any of the aforementioned complications occur, surgical correction should be pursued given the favorable outcomes. One caveat to surgical treatment, however, is the presence of cirrhosis. In these patients, studies have shown that surgical correction of abdominal hernias results in increased morbidity and mortality [12].

## Conclusions

In conclusion, hepatic herniation through the abdominal wall is a rare phenomenon that has been seldom reported in the literature. Present day epidemiologic data is lacking, but risk factors have been well established. Clinical consequences include hepatic incarceration and Budd-Chiari syndrome. These can be avoided by promptly identifying liver herniation by imaging such as CT scan, and early surgical correction on a case-by-case basis.
